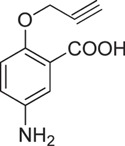# Fragment-based de novo design of a cystathionine γ-lyase selective inhibitor blocking hydrogen sulfide production

**DOI:** 10.1038/srep34398

**Published:** 2016-10-06

**Authors:** Angela Corvino, Beatrice Severino, Ferdinando Fiorino, Francesco Frecentese, Elisa Magli, Elisa Perissutti, Vincenzo Santagada, Mariarosaria Bucci, Giuseppe Cirino, Geoff Kelly, Luigi Servillo, Grzegorz Popowicz, Annalisa Pastore, Giuseppe Caliendo

**Affiliations:** 1 Department of Pharmacy, University of Naples “Federico II” – Via D. Montesano, 49, 80131–Napoli, Italy; 2 MRC Biomedical NMR Centre, NIMR, NW7 1AA, London; 3 Department of Biochemistry, Biophysics and General Pathology, Second University of Naples, Italy; 4 Technical University of Munich, Garching Campus, Munich, Germany; 5 Department of Neuroscience, Wohl Institute, King’s College London, Denmark Hill Campus, London SE5, UK

## Abstract

Hydrogen sulfide is an essential catabolite that intervenes in the pathophysiology of several diseases from hypertension to stroke, diabetes and pancreatitis. It is endogenously synthesized mainly by two pyridoxal-5′-phosphate-dependent enzymes involved in L-cysteine metabolism: cystathionine-ß-synthase (CBS) and cystathionine-γ-lyase (CSE). Research in this field is currently impaired by the lack of pharmacological tools such as selective enzymatic inhibitors that could target specifically only one of these pathways. We used a novel approach based on a hybrid method that includes drug design, synthetic biology, metabolomics and pharmacological assays to rationally design a new inhibitor selective for the CSE enzyme. The identification of this compound opens new frontiers towards a better understanding of the role of CSE over CBS in the pathophysiology of diseases where a role for the H_2_S pathway has been proposed and the development of new lead compounds that could target the CSE enzyme.

Hydrogen sulfide (H_2_S), a colorless, flammable, water soluble gas with the characteristic smell of rotten eggs, has emerged as an important gaseous signaling molecule playing numerous roles in health and disease, along with CO and NO^1^. Enzymatically generated H_2_S is mainly derived from two pyridoxal-5′-phosphate (PLP)-dependent enzymes responsible for the metabolism of L-cysteine (L-Cys): cystathionine-β-synthase (CBS) and cystathionine-γ-lyase (CSE)^2^. A third pathway that catalyses the production of H_2_S from L-Cys via the combined action of 3-mercaptopyruvate sulfurtransferase and cysteine aminotransferase has also been described^3^. This pathway is less well characterized and its role in determining the H_2_S levels in tissues still poorly understood.

CBS and CSE are cytosolic enzymes which catalyse several H_2_S-generating reactions which convert L-Cys and/or homocysteine as substrates to L-cystathionine and pyruvate respectively^4^,^5^,^6^. CBS was also originally considered to be the predominant enzyme for H_2_S production in the brain, indeed it is preferentially expressed in radial glia/astrocytes of adult and developing mouse brain^7^,^8^, whereas H_2_S synthesis in the heart and vasculature was attributed to CSE^9^. More recent studies with improved markers have provided a broader picture of enzyme distribution. Because of the co-presence of both enzymes in specific pathway it is important to find inhibitors that selectively target only one enzyme.

The most commonly used agents to inhibit H_2_S biosynthesis include propargylglycine (PAG), β-cyanoalanine (BCA) and aminooxyacetic acid (AOAA)^10^,^11^. PAG is described as CSE selective inhibitor in fact it will not inhibit recombinant CBS even if used at 10 mM^12^; moreover the crystal structure of the covalent complex PAG-CSE is the only described so far^13^. However all of these compounds have a relatively low potency and cell permeability and are used at high concentrations (mM)^11^. L-aminoethoxyvinylglycine (AVG) was also recently described as a potentially more potent and selective CSE inhibitor^12^ but its mechanism is still uncharacterised. The inherent flexibility of AVG makes it in fact difficult to rationalise the mode of binding of this compound until a crystal structure of its complex with CSE becomes available. Lack of biological data assessing the role of this compound in cell culture, tissue baths and *in vivo* further studies have also discouraged an interest on AVG, leaving PAG as the reference usually considered.

The possibility to inhibit selectively only one of these enzymes has tremendous therapeutic potentialities. CBS inhibition has for instance been suggested as a potential therapeutic to the treatment of ischemic injury. Particularly interesting would be to be able to inhibit selectively the CSE pathway since this possibility would have important consequences in several pathologies, e.g. asthma and hemorrhagic shock. PAG, for instance, has been shown to inhibit H_2_S CSE-mediated production in animal models and to accelerate recovery of blood pressure after hemorrhagic shock^14^. PAG was also shown to prevent the increase in plasma levels of markers of liver and pancreas injury, reduce the tissue content of myeloperoxidase^15^ with consequent significant prolongation of animal survival^16^. An important challenge which holds the promise to allow a much better comprehension of the H_2_S metabolism and that could be used for therapeutic purposes is thus the search for selective inhibitors able to block only one of the two enzymes and the establishment of the mechanism of interaction. However, the goal of finding such inhibitor is not an easy task given the similarity between the substrates and the mechanism involved in the enzymatic activity of the two enzymes.

We set with the aim of producing new and more selective inhibitors of CSE. Using a semi-rational *in silico* drug screening, we went through the synthesis and structural characterization of a selective CSE inhibitor. We tested the new compounds in tissue using an *ex vivo* assay with intact rat aortic rings. The compound showing maximal inhibitory effects in this test is an oxothiazolidine derivative. The effects of this compound on the enzyme kinetics were further tested *in vitro* on the purified enzymes using a metabolomics approach based on nuclear magnetic resonance techniques. By setting up complementary and more comprehensive *ex vivo* and *in vitro* assays, we also established a more accurate way to study the H_2_S pathway and demonstrated the limitations of drawing conclusions only on the basis of the methylene blue assay, the most commonly used methodology for this purpose^17^,^18^. We could conclusively prove that the identified compound has properties of a new selective inhibitor of CSE. Our results bear crucial bio-technological and pathophysiological implications which will help further studies of the H_2_S pathway.

## Results and Discussion

### Rationale of the design of the new compounds

We first carried out a preliminary *in silico* analysis of the structures of the two enzymes to understand the geometric properties of the active sites. Although both homo-tetrameric and PLP bound, their sequences and structures are very different. In CSE, essential for the reaction mechanism is a PLP covalently attached to Lys212 and stabilized by π-stacking interactions with the ring of Tyr114. In the complex with the inhibitor PAG, this is covalently bound to Tyr114 as a vinylether^13^. The amino and carboxyl groups of PAG form hydrogen bonds with Glu339 and with Arg119 and Arg62 from the adjacent monomer respectively. In CBS, PLP is bound to Lys119^19^,^20^ and is surrounded mostly by small amino acids, such as Ser349 and Asn149.

Informed by this structural analysis, we used a fragment-based approach in which we fused the structural features of the substrate cysteine with those of already known CSE inhibitors. We started by commercially available cysteine derivatives already used as substitutes of cysteine and selected L-thiazolidine-4-carboxylic acid and L-2-oxothiazolidine-4-carboxylic acid (indicated as compounds **1** and **2** in Table 1). We added to these scaffolds a propargyl group in α-carboxyl position since this structural element is essential in the proposed PAG mechanism of inhibition^13^ (compounds **1a** and **2a**). Independently, we considered the commercially available L-2-amino-3-(thiophen-2-yl)propanoic acid and 5-amino-2-hydroxybenzoic acid which were interesting because the thiazolidine moiety is substituted by aromatic groups (compounds **3** and **4**). We reasoned that an important part of the CSE active site is constituted by Tyr114 and PLP which form π-stacking interaction. The presence of an aromatic group could thus facilitate interaction of the new compound^21^. We thus attached a propargyl moiety to these scaffolds obtaining compounds **3a** and **4a**. This allowed us to have novel molecular entities which fuse the structural features of both cysteine and the CSE inhibitor PAG.

### Pharmacological assays suggest compound 2a as an inhibitor

**Figure 1:**
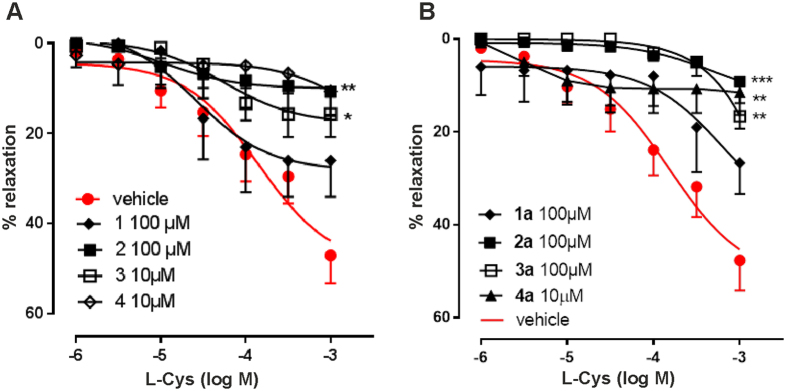
Effect of the various compounds on L-cysteine-induced vasorelaxation. (**A**) Commercially available compounds (**B**) Synthesized compounds **1a–3a** and **4a**. Each group of experiments was carried out on 4 rings harvested from different animals to obtain a more representative averaging. Vehicle, water.

The inhibitory properties of the commercially available compounds **1–4** and their newly synthesized derivatives were tested by an *ex vivo* assay on rat aortic rings by which the inhibitory activity on L-cysteine induced vasorelaxation was probed. All compounds reduced L-Cys induced vasorelaxation, with compounds **2**, **3** and **4** showing a higher inhibitory activity (for the E_max_ values refer to Suppl. Information) (Fig. 1A). Incubation of aortic rings with compound **1a** did not affect the L-cysteine induced vasorelaxation. In contrast, compound **2a** completely abrogated L-cysteine-induced vasorelaxation at a concentration of 100 μM (Fig. 1B). An inhibitory effect was observed also with compound **3a**. However, as compared to **2a**, compound **3a** was unable to completely abrogate relaxation even at the highest concentration tested (1 mM). Compound **4a**, tested at a lower concentration (10 μM) because of its limited solubility, significantly inhibited L-cysteine induced vasorelaxation. However, its solubility limits the potential use of this compound as an inhibitor and makes it difficult to assay the compound in aqueous media in a vast range of concentrations.

These results showed that only conversion of compound **2** into the propargyl derivative **2a** results in a compound with good solubility which leads to an improvement of the inhibitory activity.

### The inhibitory effect of compound 2a is direct and higher than that of known inhibitors

**Figure 2:**
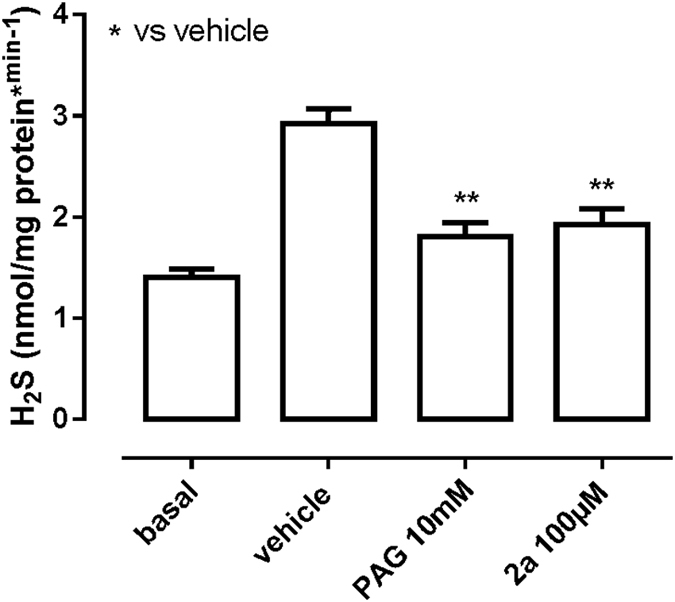
Comparison of the inhibition of compound 2a and PAG by assessment of H_2_S production in mouse aorta homogenates. The conditions for H_2_S determination were chosen according to previous protocols^17^. Homogenized aorta was incubated with different concentrations of compounds for 30 minutes and added to the reaction mixture. ZnAc (1%, 250 μL) was added to trap H_2_S followed by TCA (10%, 250 μL) and N,N-dimethyl-*p*-phenylenediamine-sulfate (20 μM, 133 μL). Absorbance was measured at a wavelength of 670 nm after 20 minutes. All samples were assayed in duplicate and compared to a calibration curve of NaHS. Basal values refers to H_2_S measurement in absence of L-Cys and it represents the endogenous production of H_2_S. Vehicle represents the used buffer, containing L-Cys.

**Figure 3:**
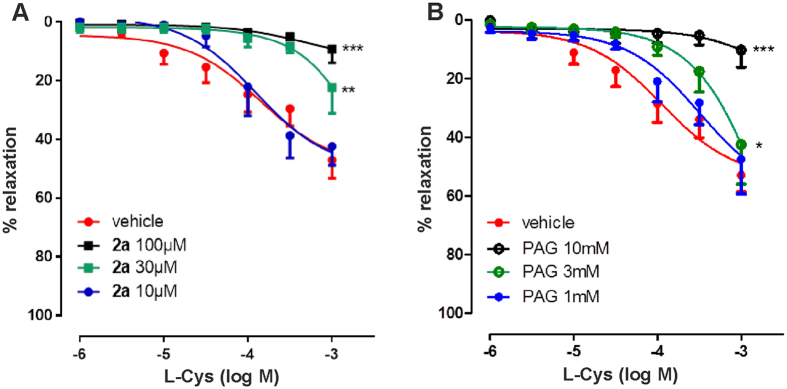
Measurement of L-Cys induced vasorelaxation. (**A**,**B**) The assay was repeated at the concentration at which **2a** and PAG exert maximal inhibition. N = 6 rings harvested from 6 different animals for each group of experiments. Vehicle, water.

Compound **2a**, which shows appreciable inhibition, was tested by the well-known methylene blue assay which measures H_2_S production (Fig. 2). Under basal conditions, tissue homogenates produced 1.4 ± 0.1% nmol of H_2_S per mg of protein. H_2_S production was stimulated by adding exogenous L-Cys into the vehicle, as routinely performed in this kind of experiment^22^,^23^. The modification doubled H_2_S production. Incubation with compound **2a** significantly inhibited the increased H_2_S production stimulated by L-Cys showing an effect comparable to that obtained with PAG but at a lower dose (100 μM for compound **2a** and 10 mM for PAG). We also compared the inhibitory activity of compound **2a** with that of PAG (Fig. 3A,B) by using isolated aortic rings.

Incubation with compound **2a** completely abrogated L-cysteine-induced vasorelaxation, while PAG reduced but not completely abrogated L-cysteine-induced vasorelaxation, despite the compound was tested at a higher concentration (10 mM and 100 μM for PAG and **2a** respectively).

To compare the efficacy of the two inhibitors, IC_50_ values were calculated determining the effect on the aorta rings relaxation in semilogarithmic scale at three different concentrations of the tested compounds. The obtained results, expressed as percent of relaxation (Fig. 3) allowed us to get the IC_50_ values. We obtained 3.3 ± 0.3 mM for PAG and 31.6 ± 0.6 μM for the compound **2a** indicating a two order magnitude improvement of the latter as compared to PAG.

These results propose compound **2a** as a more potent inhibitor than previously known ones.

To estimate the plasma membrane crossing properties of the new inhibitor we calculated the partition n-octanol/aqueous phase coefficients (Log *P*^N^ values) of the neutral form of **2a** (c log *P* = 0.27) as compared to PAG (c log *P* = −3.02) where logP is a theoretically calculated lipophilicity parameter that refers to the partition coefficient (P) in n-octanol/water of the neutral species. This tells us that the compound **2a** is more hydrophobic than PAG and this property justifies the higher concentration (10 mM) of PAG required to cross the membranes and to exert, consequently, a maximal inhibitory effect.

We also tested the importance of the propargyl group by substituting this with a propyl group which bears approximately the same volume, although having a different geometry (compound **2b**). When we tested this compound on rat aortic rings (Figure S3 of Suppl. Information), we observed no inhibitory effects indicating that the propargyl group is essential.

### Inhibition is caused by the direct and selective interaction with the CSE enzyme

The effects of the selected compound were then tested on the purified recombinant CSE and CBS enzymes as this is the only way to prove that the effects observed in *ex vivo* assays are solely the consequence of a direct and specific interaction between the compound and the enzymes. The CBS and CSE enzymes were first expressed in *Escherichia coli*, purified and characterized by SDS-PAGE, absorbance and circular dichroism techniques which confirmed that the enzymes are correctly folded and bind the respective prosthetic groups (Figure S4 of Suppl. Information).

**Figure 4:**
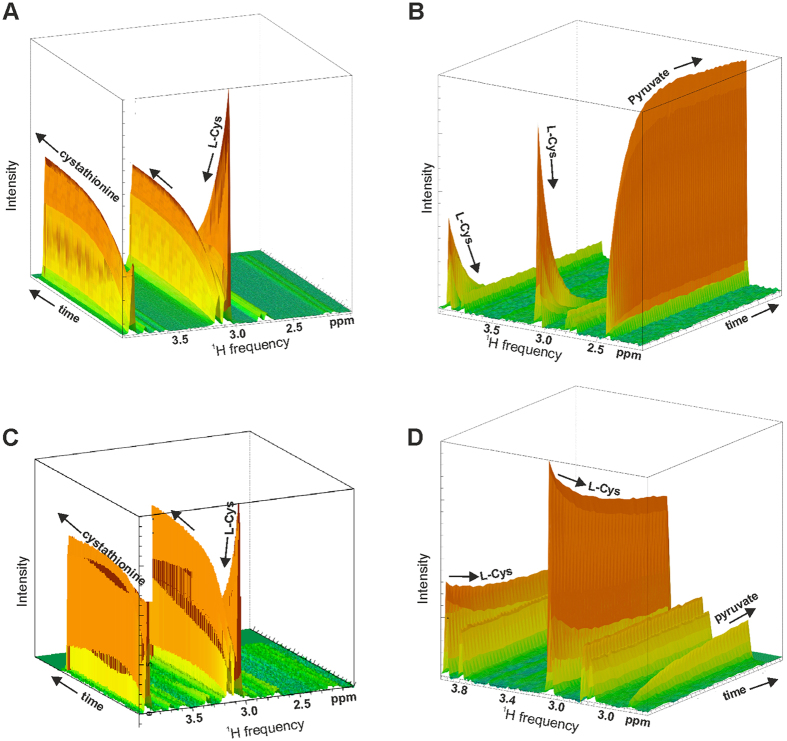
Enzymatic kinetics followed by metabolomics. Pseudo 3D plots of CBS and CSE enzymatic kinetics. The data are presented as pseudo 3D plots where the first increment of ^13^C HSQC spectra is plotted along the x (F2) and y axes. Time is plotted on the third axis. The first spectrum was obtained ~2 min after the addition of the enzyme, and subsequently 59 spectra were collected over a 300 min period. Kinetics of control isolated CBS (**A**) and CSE (**B**). Time course of CBS (**C**) and CSE (**D**) in the presence of compound **2a** (250 μM). Disappearance of the resonance at 3 ppm testifies conversion of L-cysteine to L-cystathionine by CBS and to pyruvate by CSE. The spectra were recorded at 800 MHz and 37 °C using L-Cys concentrations of 250 μM. Enzyme concentrations of 20 μM.

We used metabolomics to follow directly the conversion of L-Cys into the final products, which are respectively cystathionine and pyruvate for CBS and CSE. This strategy, which relies on high accuracy techniques such as nuclear magnetic resonance (NMR) and/or mass spectrometry (MS), has by now been vastly validated for the detection of metabolites, enzymatic products and metabolic pathways^24^,^25^. Because of its ability to identify all products rather than only specific ones, as in the methylene blue assay, metabolomics can powerfully complement these assays and provide new information. NMR spectra were recorded to detect the progressive formation of the products starting from ^15^N,^13^C-labelled substrate. For the reaction catalyzed by CBS, unlabeled L-homocysteine was also added as co-substrate. In the interest of following the kinetics at close intervals, we recorded only the first increment of ^13^C HSQC spectra at fixed time intervals (Figure S5 of Suppl. Information). Complete 2D ^13^C HSQC spectra were collected before and after each experiment to confirm completeness of the reactions. The results were best visualized as a pseudo 3D experiment in which each spectrum is plotted along the x and y axes and the third axis reflects time (Fig. 4).

In the control kinetics of CBS catalyzed production of L-cystathionine (Fig. 4A), the ^15^N,^13^C-labelled L-Cys resonances gradually decrease up to complete disappearance, while new resonances increase in intensity. The expected cystathionine resonances were not all detected, because of the unlabeled homocysteine contribution. In the control kinetics of the CSE-catalyzed reaction, the ^15^N,^13^C, L-Cys peaks decrease up to complete disappearance, while the pyruvate product increases (Fig. 4B). The presence of compound **2a** solutions pre-incubated with CBS did not alter the kinetics and the reaction proceeded with the practically complete conversion of L-Cys to L-cystathionine (Fig. 4C), whereas inhibited CSE catalyzed pyruvate production (Fig. 4D). These results conclusively prove that compound **2a** is a powerful inhibitor selective for CSE with no effects on the kinetics of CBS.

### Metabolomics reveals the limitations of the methylene blue assay

**Figure 5:**
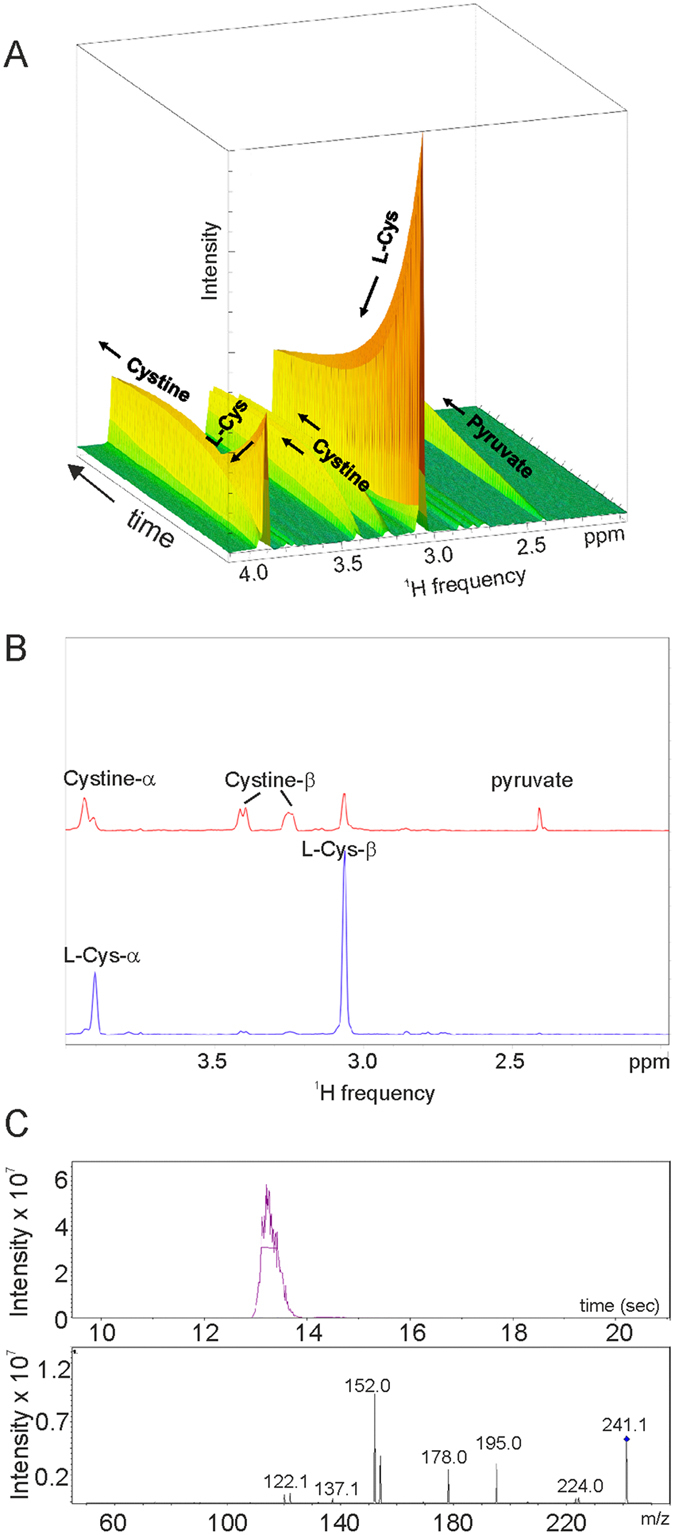
CSE kinetics in the presence of PAG followed by NMR and LC-MS. (**A)** The data are presented as pseudo 3D plots where the first increment of ^13^C HSQC spectra is plotted along the x (F2) and y axes and time is on the third axis as in Fig. 4. The concentration of PAG is 250 μM. (**B**) The first (blue) and last (red) increments of a ^13^C HSQC. The first spectrum was obtained ~2 min after the addition of the enzyme, and subsequently 59 spectra were collected over a 300 min period. The conditions used are the same as in Fig. 4. (**C**) Extracted ion chromatogram of CSE reaction mixture in the present of PAG at the end of the reaction, carried out for 60 minutes, by adding L-cysteine and incubating the mixture at 37 C. The chromatography was performed by monitoring the MS/MS transition 241.1−>152.0. The panel below the chromatogram reports the MS/MS fragmentation pattern of the peak at retention time of 13.5 min, which corresponds to cystine. The injection volumes were 10–20 μL for each sample.

During our NMR based metabolomics studies we observed something peculiar: when PAG was used in the NMR assay, no appreciable formation of pyruvate was observed, as expected, but the resonances of L-Cys surprisingly decreased and new product resonances, different from pyruvate, appeared and progressively increased (Fig. 5A). This behaviour was also confirmed in a ^13^C 2D HSQC spectrum acquired at the end of the kinetics (Fig. 5B): in the presence of PAG, the cysteine resonances at 3.90 ppm (C_α_H and 55 ppm in the carbon spectrum not shown) and at 3.05 ppm (C_β_H_2_ and 25 ppm) were absent at the end of the reaction. This means that, although PAG inhibits H_2_S formation, it is not able to block cysteine consumption. This is *per se* an important result which warns us against the inefficacy of PAG. New peaks at 3.92 ppm, 3.40 ppm and 3.25 ppm appeared instead (and at 55 ppm, 32 ppm and 32 ppm in the carbon spectrum respectively). These chemical shifts were tentatively assigned to cystine, lanthionine or a similar compound. To confirm which of these compounds is the additional product we used the high accuracy LC-MS technique^25^ (Fig. 5C). We found that, during the reaction, a predominant quantity of cysteine is converted into cystine with a minor quantity of lanthionine. Identification of this reaction product has so far been overlooked likely because all previous studies solely relied on the methylene blue assay which allows only detection of H_2_S production. Since the presence of an excess of freshly prepared DTT makes it unlikely chemical formation of cystine by oxidation, a possible explanation is that cystine is formed enzymatically.

### *In vitro* assays suggest that the new inhibitor is not suicidal and competitive

We characterized the complex of CSE with compound **2a** by mass spectrometry to test whether also this inhibitor, like PAG, is suicidal. We found no increase of the mass of CSE suggesting no covalent attachment of compound **2a** (Figure S6 of Suppl. Information). This result agrees with the suggested mechanism of inhibition which postulates the need of a NH_2_ group for the reaction to occur^13^. To quantify the inhibitory potency towards CSE of compound **2a** with respect to PAG, we used an adapted version of a previously described spectrophotometric assay^17^. The IC_50_ measured on the purified protein is 6.3 ± 1.0 μM for **2a** versus 42.2 ± 1.1 μM for PAG (Figure S7 of Suppl. Information). This value is in excellent agreement with the data from the *ex vivo* assay.

**Figure 6:**
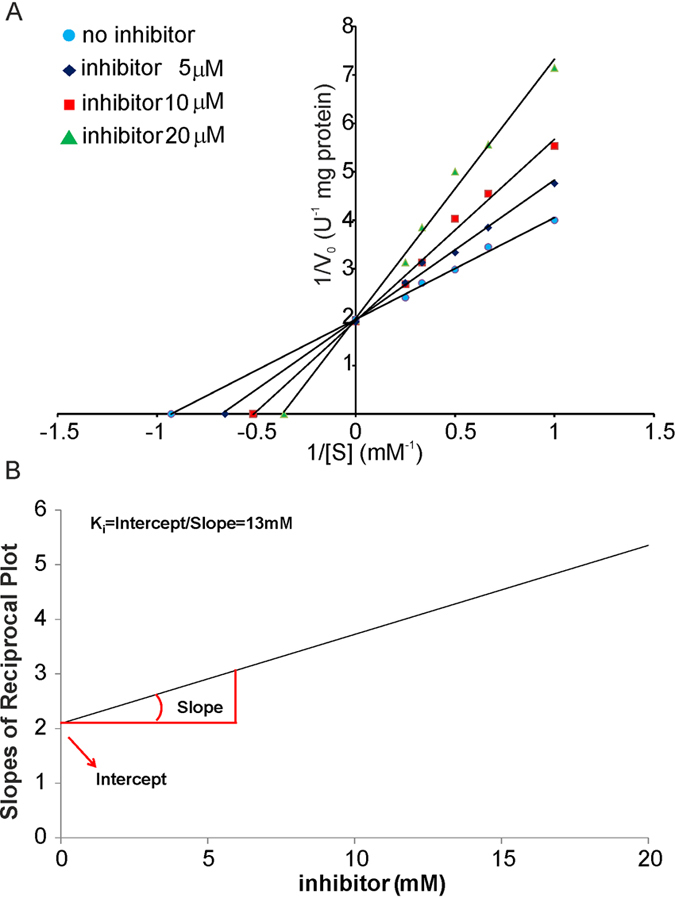
Enzymatic characterization of the effects of compound 2a on purified CSE. (**A)** Lineweaver–Burk plot of CSE catalyzed H_2_S production in the absence (control) and presence of various concentrations of compound **2a** (5 μM, 10 μM and 20 μM). The data suggest that 2a is a competitive inhibitor, since the plots are linear and intersect at the y-axis (K_m_ increases while V_max_ remains unaffected). For reference, non-competitive inhibition would produce plots with the same *x*-intercept as uninhibited enzyme (*K*_*m*_ is unaffected) but different slopes and *y*-intercepts. Uncompetitive inhibition would cause different intercepts on both the *y*- and *x*-axes. The estimated V_max_ and K_m_ values are 0.50 ± 0.02 U/mg and 1.1 ± 0.1 mM. The K_m, apparent_ values are 1.5 ± 0.1 mM, 1.9 ± 0.3 mM and 2.8 ± 0.3 mM for the data at 0 mM, 5 μM, 10 μM and 20 μM of inhibitor, respectively. (**B**) Slopes of the double reciprocal plot *vs.* the concentrations of inhibitor (0, 5, 10 and 20 μM) (Fig. 6B). The intercept gives the value of K_m_/V_max_ and the slope is K_m_/(V_max_ K_i_). K_i_ is obtained as the intercept/slope ratio.

With the same spectrophotometer assay described above, we also determined the Michaelis-Menten constants V_max_, the maximum reaction rate at maximum saturation of the substrate concentration, and K_m,_ the substrate concentration at which the reaction rate is half of V_max_, at different concentrations (0–20 μM) by nonlinear regression analysis using the Michaelis-Menten equation (Fig. 6A). The K_m_ value is 1.1 ± 0.1 mM, instead the K_m,apparent_ values at various concentrations of compound **2a** (5 μM, 10 μM and 20 μM) are 1.5 ± 0.1 mM, 1.9 ± 0.3 mM and 2.8 ± 0.3 mM, respectively. The V_max_ is 0.50 ± 0.02 U/mg at all the tested concentrations of the inhibitor. These kinetic parameters suggest that compound **2a** is a competitive inhibitor of the enzyme. Furthermore, the slopes from the double reciprocal plot are plotted in a secondary plot *vs.* the concentrations of inhibitor used (Fig. 6B). The intercept and the slope of this graph is K_m_/V_max_ and K_m_/(V_max_ K_i_), respectively, thus, the K_i_ is 13 μM.

While a more thorough enzymatic characterization will be needed, these data suggest the mechanism of action of the new inhibitor which has quite distinct properties from PAG.

### Rationalizing the results

**Figure 7:**
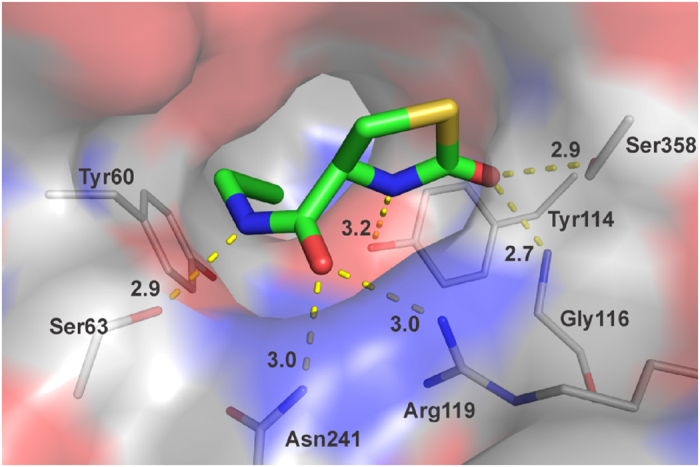
Structural model of compound **2a** (stick representation) docked in the coordinates of CSE (transparent surface and side chains of nearby residues) in complex with PAG (not shown here). A space-filling representation is used for the enzyme surface, coloured according to the electrostatic charge. The compound is shown with a stick representation. The enzyme structure (3cog) was drawn with the graphic software MAESTRO.

We rationalized our findings analysing the structure of CSE. Starting from the coordinates of CSE (PDB code 3cog) which contain the covalently bound PAG, we built in manually the coordinates of compound **2a** using the software MAESTRO (Schroedinger, LLC, New York, NY). The cavity which contains PAG in the active site, which is also where the substrate cysteine should bind, is big enough to accommodate also compound **2a** (Fig. 7).

This can easily form multiple interactions with the surrounding groups and hydrogen bond with the side chains of Ser63, Tyr114, Gly116, Arg119, Asn241, and Ser358. Amongst these interactions, particularly important would be those formed by the carbonyl group of the L-2-oxothiazolidine moiety which could form a bifurcated hydrogen bond with Gly116 and Ser358. The triple bond would occupy a position similar (<2A difference) to that of PAG without being covalently bound and could not easily be replaced because the pocket between the two tyrosines is very deep and narrow. A triple bond forms a long thin “rod” of very small van der Waals radius which could thus fit in, while double or single bonds could not be allocated between the tyrosines for lack of space. Pi-stacking of the triple bond with the two tyrosine rings could additionally contribute to the stability of the interaction.

This configuration would also explain why compounds **1** and **1a** do not fit: they have no carboxy group which makes the ring to be bent while it is quite flat in **2a** and this prevents the possibility of hydrogen bonding to the carbonyl oxygen. The bent ring will most likely not fit in the pocket. Compounds **3** and **3a** have a CH_2_ linker which positions the ring too far from the pocket to form interactions and changes its angle relative to the triple bond. Compounds **4** and **4a** are simply too big for the pocket.

We can thus conclude that we have identified a new compound which has all features to optimally fit in CSE and inhibit selectively its function.

## Conclusion

Investigation of the pathophysiological and pharmacological roles of hydrogen sulfide has rapidly become an important research field still widely unexplored which is attracting increasing attention. H_2_S is now accepted as a novel gasotransmitter of potential importance in several areas from asthma to cancer, impotence and stroke^26^. A major obstacle in the rapid development of this field has however been the lack of pharmacological tools such as selective enzymatic inhibitors which would allow the study of only one among the several possible pathways. The already established inhibitors exhibit low potency and selectivity. We have thus aimed at developing more specific and potent inhibitors of CBS and CSE towards H_2_S production. We used a semi-rational strategy which combines two highly successful routes, the structural based and the fragment-based approaches and went all way from drug design to *ex vivo* and *in vitro* studies. We identified an oxothiazolidine derivative which showed maximal inhibitory effects *in tissue* and proved to be more efficient than PAG and completely selective for CSE by metabolomics assays on the purified enzymes. As compared to PAG, the new compound has a significant difference: both compounds inhibit CSE from producing pyruvate, ammonia and hydrogen sulfide, but surprisingly, in the presence of PAG, CSE remains able to consume the substrate L-Cys and produces cystine and lanthionine. This is an important result previously under looked. In contrast, compound **2a** inhibits the enzyme completely, keeping the concentration of substrate unaffected. This finding suggests the possibility of using lower doses of compound **2a** compared to PAG, consequently also reducing all possible adverse effects. PAG is also a suicidal inhibitor while **2a** does not bind irreversibly offering a further advantage.

In conclusion, it is clear that before using the new compound for human treatment, it will be essential to carry out a careful pharmacological study to assess whether the new compound is toxic or is associated with any unwanted side effects. For the time being, we are nevertheless confident that identification of this highly selective CSE inhibitor will help to better define the role of CSE vs CBS in the pathophysiology of the diseases where a role for the H_2_S pathway has been proposed. Furthermore, the development of such agents, particularly water-soluble, will allow us to evaluate the cross-talk of H_2_S pathways with other relevant pathways (e.g. NO, COX).

## Experimental section

### Compound synthesis and their identification

The synthesis strategy is detailed below and summarized in Figures S1 and S2 of Suppl. Information. The identity of the resulting products was proven using mass spectrometry, ^1^H and ^13^C NMR. All compounds resulted correctly synthesized. Melting points, determined using a Buchi Melting Point B-540 instrument, are uncorrected and represent values obtained on recrystallized or chromatographically purified material. ^1^H-NMR spectra for compound characterization were recorded on a Varian Mercury Plus 400 MHz instrument and in DMSO solutions. Chemical shifts are reported in ppm using Me_4_Si as internal standard. The following abbreviations are used to describe peak patterns when appropriate: s (singlet), d (doublet), t (triplet), q (quartet), qt (quintet), dd (double doublet), m (multiplet). Mass spectra of the final products were performed on API 2000 Applied Biosystem mass spectrometer. Elemental analyses were carried out on a Carlo Erba model 1106. Analyses indicated by the symbols of the elements were within ±0.4% of the theoretical values. All reactions were followed by TLC, carried out on Merck silica gel 60 F254 plates with fluorescent indicator and the plates were visualized with UV light (254 nm). Preparative chromatographic purifications were performed using silica gel column (Kieselgel 60). Solutions were dried over Na_2_SO_4_ and concentrated with a Buchi R-114 rotary evaporator at low pressure.

### Isolated mouse aortic ring assay

The animals were sacrificed according to the regulations of University of Naples. Thoracic aorta from CD-1 mice was rapidly dissected and cleaned from fat and connective tissue. Animal care was in accordance with Italian and European regulation on the protection of animals used for experimental and other scientific purposes. For endothelium-denuded rings, the endothelial layer was removed by gently rubbing the internal surface of the vascular lumen. Rings of 1.5–2 mm length were cut and mounted in isolated organ baths filled with gassed Krebs solution containing (in mol/L) NaCl 0.118, KCl 0.0047, MgSO_4_ 0.0023, KH_2_PO_4_ 0.0012, CaCl_2_ 0.0025, NaHCO_3_ 0.025 and Glucose 0.010) (95% O_2_ + 5% CO_2_) at 37 °C linked to isometric force transducers (UgoBasile, Comerio, Varese, Italy). Rings were initially stretched until a resting tension of 1.5 g was reached and equilibrated for at least 45 min during which tension was adjusted to 1.5 g. The bathing solution was periodically changed. In each experiment, rings were contracted with L-phenylephrine (PE) at 3 μmol/L and then at 1 μmol/L until response was reproducible. To verify the integrity of the endothelium, aortic rings were contracted with PE (1 μM), once plateau was reached and a cumulative concentration-response curve to commercially available precursors or the newly synthesized compounds (10 nM – 300 μM) was performed. The rings were contracted with PE (1 μM) and a cumulative concentration-response curve to L-cysteine (1 μM - 3 mM) was performed. All data are presented as means ± SEM. Statistical analysis was performed by two-way ANOVA followed by Bonferroni multiple comparison test. Differences were considered statistically significant when P-value was <0.05. GraphPad Prism software (GraphPad Software, Inc., La Jolla, CA, USA) was used for all the statistical analysis. IC_50_ values were determined *ex vivo*, using at least three different concentrations of the tested compounds. All the experiments were performed in triplicate. The obtained data, expressed in semilogarithmic scale, were used by the ED_50_ Plus v1.0 online software to get the IC_50_ values. All experimental protocols were approved by the licencing committee of University of Naples.

### Estimate of the Lipophilic parameters

To estimate the plasma membrane crossing properties of the new inhibitor, the Log *P*^N^ values, i.e. partition coefficients n-octanol/aqueous phase of the neutral form of selected compounds, were calculated (c log*P*) by the program *C*logP for Windows version 2.0 (Biobyte Corp., Claremont, CA).

### Measurement of H_2_S production: methylene blue assay

H_2_S determination was performed, *ex vivo* and *in vitro*, modifying previous protocols^17^.

For the *ex vivo* assay, thoracic aorta was dissected, placed in sterile phosphate buffer solution and cleaned of fat and connective tissue. The aortic rings were homogenized in a lysis buffer (potassium phosphate buffer 100 mM pH 7.4, sodium orthovanadate 10 mM and protease inhibitor) and treated with the different concentrations of compounds for 30 minutes and added in a reaction mixture (total volume 500 μL) containing piridoxal-5’-phosphate (2 mM, 20 μL), L-cysteine (10 mM, 20 μL) and saline (30 μL). The reaction was initiated by transferring tubes from ice to a water bath at 37 °C. ZnAc (1%, 250 μL) was added to trap evolved H_2_S followed by TCA (10%, 250 μL) and N,N-dimethyl-*p*-phenylenediamine-sulfate (20 μM, 133 μL) in 7.2 M HCl and FeCl_3_ (30 μM, 133 μL) in 1.2 M HCl were added. After 20 minutes absorbance values were measured at a wavelength of 670 nm. All samples were assayed in duplicate and H_2_S concentration was calculated against a calibration curve of NaHS (3.12–250 μM). Basal release of H_2_S was measured in absence of L-Cys. Vehicle indicates the used buffer, containing exogenous L-Cys.

For the *in vitro* assay, each reaction mixture (250 μL) consisted of 5 μg of human CSE, 0.01 mM PLP, 1 mM L-cysteine and 50 mM sodium phosphate pH 8.2 buffer. The inhibitors, at different concentrations, were added to each reaction 15 min before L-cys was added to the solution. Reaction was initiated by transferring the Eppendorf tubes, tightly parafilmed, from ice to a 37 °C water bath. After 30 min of incubation, the evolved H_2_S was trapped via addition of ZnAc (1% w/v, 100 μL) and proteins were precipitated via addition of TCA (10% w/v, 100 μL). Subsequently, N,N-dimethyl-p-phenylenediamine-sulfate in 7.2 M HCl was immediately followed by addition of FeCl_3_ in 1.2 M HCl, for development of methylene blue. The amount of H_2_S produced was determined via absorbance measurements of the centrifugal supernatants. The absorbance of the resulting solution was measured at 670 nm and the amount of H_2_S was calculated against a calibration curve of standard H_2_S solutions.

The determination of IC_50_ on purified CSE was done by a concentration-response curve obtained by plotting the percentage of inhibition versus the logarithm of inhibitor concentrations. The IC_50_ values were estimated from the resulting graph using GraphPad Prism v.6.07 (GraphPad Software, Inc. La Jolla, CA).

### Protein expression and purification

E. coli BL21 (DE3) Codon Plus was used as the host strain to express recombinant human CSE or CBS. CSE cDNA was cloned into pGEX-4T3 and CBS into pGEX-Kg to create N-terminal GSH-S-transferase (GST) fusion proteins. The expression vectors were transformed and plated on LB-agar plates, supplemented with ampicillin (100 mg·mL^−1^). The expression and purification of CBS and CSE was adapted from previously described protocols^21^. Colonies of pGEX-Kg/GST-CBS or pGEX-4T3/GST-CSE transformed BL21 cells were separately inoculated into 100 mL of steam-autoclaved Luria-Bertani (LB) broth supplemented with 100 μg/mL of ampicillin (LB-Amp100), and incubated at 37 °C overnight shaking. After induction and harvesting, the lysates were centrifuged at 16000 rpm for 30 min at 4 °C using a Beckman JA20 rotor. The soluble fractions were loaded onto Glutathione sepharose 4B columns equilibrated with binding buffer PBS. The flow-throughs were collected, and the beads washed. Proteins attached to the columns were eluted with five column volumes of elution buffer (50 mM Tris–HCl, 10 mM reduced GSH, 5mM DTT, pH 8.0), dialyzed and concentrated in 10 mM sodium phosphate buffer pH 8.2 and DTT 1 mM. The eluates were passed through a Superdex-200 column connected to the AKTA purifier FPLC equilibrated in the same buffer at a flow rate of 1 mL/min. Fractions containing the enzymes were identified by sodium dodecyl sulfate polyacrylamide gel electrophoresis (SDS-PAGE), pooled together and concentrated using an ultra-centrifugal filter (Millipore Amicon Ultra-4 10000 MWCO) at 4 °C.

### Structural characterization of recombinant enzymes

Far-UV CD measurements were performed on a J-715 spectropolarimeter (JASCO, Oklahoma City, OK) equipped with a PTC-348 Peltier temperature control system using an enzyme concentration of 4 μM in 10 mM sodium phosphate pH 8.2 buffer. The spectra were recorded in quartz cuvettes (Hellma, Plainview, NY) with path-lengths of 1 mm and averaging five separate wavelength scans on independent protein preparations to ensure reproducibility. Baseline correction was performed by subtraction of the appropriate buffer spectrum. Adsorbance spectroscopy was carried out on a Varian Cary 50 Bio UV-Visible Spectrophotometer. Measurements were performed in a 1 cm cuvette cell and monitored spectroscopically from 650nm to 250 nm.

### Enzymologic analysis

Kinetics studies were performed using an adapted version of a spectrophotometric assay described by Stipanuk and Beck^17^. Assay mixtures (250 μL) contained 5 μg of purified CSE, 2 mM PLP and L-cysteine (1–4 mM) previously incubated at 37 °C. For each L-cysteine substrate concentration, the amount of H_2_S was measured every 3 min over 30 min. Zinc acetate (1% w/v) was added to trap the produced H_2_S gas. The enzymatic reaction was stopped by the addition of 10% (w/v) trichloroacetic acid. N,N-dimethyl-p-phenylenediamine dihydrochloride dye (20 mM) and 30 mM FeCl_3_ were added and the absorbance at 670 nm was measured using a microplate reader (Thermo Scientific). All determinations were carried out in triplicates. The amount of H_2_S produced was calculated against a calibration curve of sodium hydrosulfide (0–250 μM). The initial reaction velocity, V_0_ (units/mg of human CSE, where 1 unit is the amount of enzyme that catalyses the reaction of 1 μmol of H_2_S per minute) was plotted against the cysteine substrate concentration [S]. Rates were fit with the Michaelis-Menten equation using GraphPad Prism software version 7 for Windows (San Diego, USA).

### NMR spectroscopy

Samples were composed of ^15^N,^13^C-L-cysteine (125 μL, 1 mM), unlabeled L-homocysteine (125 μL, 1 mM) (added only for the CBS time course as co-substrate), DTT (0.5 μL, 1M), D_2_O (50 μL), and enzyme (100 μL, 110 μM) in 10 mM sodium phosphate pH 8.2 and filled to a final volume of 500 μL. NMR experiments were performed at 300 K on a Bruker AVANCE spectrometer operating at 800 MHz with TCI Cryoprobes. Controls were run excluding either the enzyme or the tested compounds pre-incubated with each enzyme. For each sample, pseudo-2D experiments were collected in which the first increment of a ^13^C HSQC spectrum was acquired with 5 minutes repetition time for a total of 60 spectra, using ^15^N,^13^C, L-cysteine and unlabeled L-homocysteine as CBS substrate and ^15^N,^13^C, L-cysteine as a unique substrate for CSE enzyme. 2D ^1^H,^13^C HSQC spectra were acquired before and after the enzyme reaction was completed.

### HPLC-ESI-MS/MS Analyses

Chromatographic analyses were performed using a HPLC Agilent 1100 series equipped with an auto-sampler coupled online with an Agilent LC-MSD SL quadrupole ion trap. The liquid chromatography separation was carried out on a Supelco Discovery-C8 column (250 × 3.0 mm, particle size 5 μm) eluted isocratically with 0.1% formic acid in water, at a constant flow rate of 0.1 mL/min. The injection volumes were 10–20 μL for each sample. Electrospray ionization (ESI) was in positive ion mode, with nitrogen as the nebulizing and drying gas using a pressure of 30 psi, a drying temperature of 350 °C and drying gas at 7 L/min. The ion charge control (ICC) was applied with the target set at 30000 and maximum accumulation time at 20 ms. Optimization of instrumental parameters was performed by continuous infusion of 5 μM standard solution in 0.1% formic acid. Mass cutoff and amplitude were optimized to obtain the most efficient MS/MS transitions from the positively charged precursor ion [M + H]^+^ to the fragment ions. Multiple reaction monitoring (MRM) was used with the MS/MS transitions utilized for the quantitation of analytes of 209.1→120 for lanthionine, 241.0→152 for cystine, 122.0→105 for cysteine. Quantitation was achieved by comparison with calibration curves obtained from standard solutions. Measurements were performed from the peak area of the extracted ion chromatogram (EIC). Retention times and peak areas of the monitored fragment ions were determined by the Agilent software Chemstation version 4.2.

### Structural analysis

Structural analysis and manual docking was possible with the support of the software PYMOL and MAESTRO (Schroedinger, LLC, New York, NY). The position of PAG in the X-ray structure 3cog was taken as pose of reference.

## Additional Information

**How to cite this article**: Corvino, A. *et al*. Fragment-based de novo design of a cystathionine γ-lyase selective inhibitor blocking hydrogen sulfide production. *Sci. Rep.* **6**, 34398; doi: 10.1038/srep34398 (2016).

## Supplementary Material

Supplementary Information

## Figures and Tables

**Table 1:** Chemical formulas of the compounds commercially available (left two columns) and synthesized for the present work (right two columns).

Compd	Structure	Compd	Structure
**1**	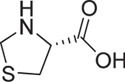	**1a**	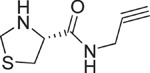
**2**	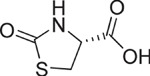	**2a**	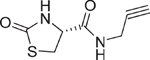
**3**	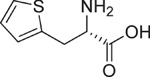	**3a**	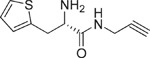
**4**	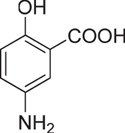	**4a**