# Supplementary material for: Fragment-based de novo design of a cystathionine γ-lyase selective inhibitor blocking hydrogen sulfide production
Source: Sci Rep. 2016 Oct 6;6:34398. doi: 10.1038/srep34398 (PMC5052628; doi:10.1038/srep34398)
Supplement: Supplementary Information [file srep34398-s1.doc]

**Supporting Information**

**Fragment-based de novo design of a cystathionine γ-lyase selective inhibitor blocking hydrogen sulfide production**

Angela Corvino^†^, Beatrice Severino^†^, Ferdinando Fiorino^†^, Francesco Frecentese^†^, Elisa Magli^†^, Elisa Perissutti^†^, Vincenzo Santagada^†^, Mariarosaria Bucci^†^, Giuseppe Cirino^†^, Geoff Kelly^§^, Luigi Servillo^++^, Grzegorz Popowicz^#^, Annalisa Pastore^‡*^ and Giuseppe Caliendo^†*^

^†^Department of Pharmacy, University of Naples “Federico II” – Via D. Montesano, 49, 80131 –Napoli, Italy

^§^MRC Biomedical NMR Centre, NIMR, London, NW7 1AA

^++^Istituto di Biochimica, Biofisica e Patologia Generale, Seconda Universita’ degli Studi di Napoli, Italy

^#^Technical University of Munich, Garching Campus, Munich, Germany

^‡^Department of Neuroscience, Wohl Institute, King's College London, Denmark Hill Campus, London SE5, UK

The synthetic route for the preparation of propargylamide derivatives (**1a-3a**) is reported in **Figure S1**. The carboxylic acid was treated with N-(9-fluorenylmethoxycarbonyloxy) succinimide to protect the amine group, when needed. The coupling reaction of N-protected carboxylic acid with propargylamine hydrochloride was performed in the presence of N,N,N′,N′-Tetramethyl-O-(benzotriazol-1-yl)uronium tetrafluoroborate (TBTU), N-Hydroxybenzotriazole (HOBt) and N,N-diisopropylethylamine (DIPEA), leading to the formation of an amide bond. Fmoc deprotection, when necessary, was carried out using a solution of 33% DIEA in tetrahydrofuran, obtaining compounds **1a-3a**.

**Figure S1**

The synthesis of compound **4a** (**Figure S2**) includes protection of amine and carboxyl groups, ether formation using the conventional Williamson synthesis, by treatment with NaH and propargyl bromide performed in anhydrous CH_3_CN; subsequent Boc deprotection and methyl ester hydrolysis. The propyl derivative **2b** was prepared as for compound **2a** employing propylamine instead of propargylamine hydrochloride.

**Figure S2**

*Preparation of N-(prop-2-yn-1-yl) thiazolidine-4-carboxamide (1a)*

The thiazolidine-4-carboxylic acid (**1**, 1 g, 7.5 mmol) in Na_2_CO_3_ 9% (10 mL) was cooled in ice water and mechanically stirred. A solution of Fmoc-OSu (2.5 g, 7.5 mmol) and dioxane (20 mL) was added dropwise and the mixture was stirred for 2.5 hours at room temperature. The solvent was evaporated, the aqueous residue acidified with 1N HCl and the product extracted with ethyl acetate. The organic phase was dried on anhydrous Na_2_SO_4_ and filtered, and the solvent was evaporated. The product was crystallized from diethyl ether, yielding 2.5 g of 3-(((9H-fluoren-9-yl)methoxy)carbonyl)thiazolidine-4-carboxylic acid (**5**) as a solid.

The intermediate **5** (1 g, 2.8 mmol) was dissolved in DMF (30mL) and coupled to propargylamine hydrochloride (0.258 g, 2.8 mmol), in the presence of DIPEA (0.536 mL, 3.1 mmol), using HOBt (0.472 g, 3.1 mmol) and TBTU (0.988 g, 3.1 mmol) as the coupling reagents. This reaction was carried out at room temperature with stirring overnight. The solvent was evaporated, and the crude material was dissolved in ethyl acetate (150 mL) and washed with 5% citric acid (3 x 50 mL), 10% NaHCO_3_ (3 x 50 mL) and brine (50 mL). The organic layer was dried on anhydrous Na_2_SO_4_ and filtered, and the solvent was evaporated. After chromatography on a silica gel column (eluent, 7:3 ethyl acetate/hexane) the intermediate **7** was obtained as a solid. Fmoc deprotection of intermediate **7** was performed using a 33% diethylamine solution in tetrahydrofuran and the mixture was stirred for 2.5 hours at room temperature. Afterward the solvent was concentrated *in vacuo* and the obtained residue was purified by column chromatography (dichloromethane/methanol 9.5:0.5 (v/v)). The combined and evaporated product fractions were crystallized from hexane, to give 330 mg of the final compound N-(prop-2-yn-1-yl)tiazolidin-4-carboxamide **1a** (69% calculated on two synthetic steps) as white solid.mp 126−127 °C; ^1^H NMR (400 MHz, DMSO-d_6_) δ 3.13 (s, 1H, CCH),3.10-3.18 (dd, 1H, CH_2_CH thiaz, J=6.65), 3.39-3.43 (dd, 1H, CH_2_CH thiaz, J=6.65),3.88 (s, 2H, NHCH_2_), 3.96 (d, 1H, SCH_2_NH, J=9.90), 4.13 (t, 1H, CH_2_CHthiaz), 4.24 (d, 1H, SCH_2_NH, J=9.90), 8.29 (s, 1H, NH), 8.99 (s, 1H, NH); ^13^C NMR (400 MHz, DMSO-d_6_) δ 29.0, 32.9,61.8, 74.0, 74.6, 81.3,170.1; ESI-MS calculated for C_7_H_10_N_2_OS 170.23; found (M + H)^+^ 171.00.

*Preparation of 2-oxo-N-(prop-2-yn-1-yl) thiazolidine-4-carboxamide (2a)*

A mixture of 2-oxo-N-thiazolidine-4-carboxilic acid **2** (1 g, 6.8 mmol), propargylamine hydrochloride (0.622 g, 6.8 mmol), HOBt (1.15 g, 7.5 mmol), TBTU (2.40 g, 7.5 mmol) and DIPEA (1.30 mL, 7.5 mmol) in DMF (20 mL) was stirred overnight at room temperature. The solvent was concentrated to dryness and the crude mixture was purified by silica gel column chromatography using ethyl acetate/hexane 7:3 (v/v) as eluent. The crude product was recrystallized from hexane, yielding 0.96 g of 2-oxo-N-(prop-2-yn-1-yl)thiazolidine-4-carboxamide **2a** (76%): mp 131.5−132.4 °C; ^1^H NMR (400 MHz, DMSO-d_6_) δ 3.13 (s, 1H, CCH), 3.62-3.68 (dd, 2H, CH_2_thiaz, J=8.61), 3.88 (s, 2H, NHCH_2_), 4.27 (t, 1H, CH_2_CHthiaz), 8.27 (s, 1H, NH), 8.53 (s, 1H, NH); ^13^C NMR (400 MHz, DMSO-d_6_) δ 29.0, 32.9, 56.8, 74.0, 81.3, 170.6, 174.0; ESI-MS calculated for C_7_H_8_N_2_O_2_S 184.22; found (M + H)^+^ 184.90.

*Preparation of 2-amino-N-(prop-2-yn-1-yl)-3-(thiophen-2-yl) propanamide (3a)*

The desired product **3a** has been obtained following the same procedure previously reported for compound **1a** starting from the L-2-amino-3-(thiophen-2-yl)propanoic acid (**3**), suitably protected, coupled to propargylamine hydrochloride (0.229 g, 2.5 mmol) and subsequently deprotected. Yield 68% (calculated on two synthetic steps): mp196.2-196.3°C; ^1^H NMR (400 MHz, DMSO-d_6_) δ 3.18 (s, 1H), 3.27-3.31 (dd, 2H, CH_2_CH, J=6.06), 3.90 (s, 2H, NHCH_2_), 3.95 (t, 1H, CHCO, J=6.09), 6.94-6.97 (m, 2H, H-thioph), 7.40 (d, 1H, H-thioph, J=3.9), 8.38 (s, 2H, NH), 9.05 (s, 1H, NH_2_); ^13^C NMR (400 MHz, DMSO-d_6_) δ 28.9, 31.4, 53.9, 74.6, 80.7, 126.4, 127.9, 128.4, 136.4, 168.0; ESI-MS calculated for C_10_H_12_N_2_OS 208.28; found (M + H)^+^ 209.20.

*Preparation of 5-amino-2-(prop-2-ynyloxy)benzoic acid (4a)*

To a solution of 5-amino-salicylic acid (**4**, 2 g, 13.0 mmol) in 25 ml of dioxane and 12.5 mL of water, triethylamine (2.6 mL, 19.5 mmol) and di-*tert*-butyl-dicarbonate (4.2 g, 19.5 mmol) were added with stirring at 0° C for 1/2 h. The reaction mixture was stirred mechanically for 24 h at room temperature. After evaporation of the solvent, 3N HCl (15 mL) was added dropwise to the residue. The precipitate is filtered, washed with water, dried and recrystallized from diethyl ether. 5-(*tert*-butoxycarbonylamino)-2-hydroxybenzoic acid was obtained (**9**) (3.28 g, 100 % yield) and the powder (3 g, 11.8 mmol) was dissolved in anhydrous methanol. To the solution, cooled to 0 °C, dicyclohexylcarbodiimide (2.4 g, 11.8 mmol) was added and the mixture was stirred at room temperature for 3h. The solvent was evaporated, the residue was taken up in ethyl acetate and dicyclohexylurea (DCU) was filtered. After chromatography on a silica gel column (eluent, 7:3 ethyl acetate/hexane) 2.3 g of **10** was obtained as a white solid. Intermediate **10** (1 g, 4.0 mmol) was dissolved in anhydrous acetonitrile and treated with potassium carbonate (0.475 g, 4.0 mmol) and propargyl bromide (solution 80% wt. in toluene) (0.446 mL, 4.0 mmol) was added slowly to obtain the propargyl derivative (**11**). The reaction mixture was stirred under reflux overnight. After the solvent was removed, the residue was purified by column chromatography (3:7 ethyl acetate/hexane (v/v)). The combined and evaporated product fractions were crystallized from diethyl ether/hexane, affording methyl 5-(*tert*-butoxycarbonylamino)-2-(prop-2-ynyloxy)benzoate (**11**).To a solution of **11** (0.734 g, 2.5 mmol) in ethanol, sodium hydroxide was added and the mixture was stirred at room temperature for 2 hours. The solvent was removed *in vacuo* and residue was slightly acidified with 1N HCl. The aqueous solution was extracted with ethyl acetate and the organic phase was dried over anhydrous Na_2_SO_4_, filtered and evaporated to dryness to afford the compound **12** as a white powder. The crude intermediate (**12**, 0.734 g, 2.5 mmol) was treated with a solution of 40 % TFA in DCM. After 2 h the solvent was removed to obtain the crude product. The residue was loaded on a silica gel open column and eluted with ethyl acetate/hexane (7:3 v/v).The combined and evaporated fractions were crystallized from hexane to yield the pure product **4a**. Yield 87%: mp 220.0-220.4°C; ^1^H NMR (400 MHz, DMSO-d_6_); δ 3.48 (s, 1H, CH), 4.63 (s, 2H, CH_2_), 6.69 (d, 1H, J=8.43, Ar-H), 6.89 (d, 1H, J=8.43, Ar-H), 6.91 (s, 1H, Ar-H); ^13^C NMR (400 MHz, DMSO-d_6_) δ 58.43 78.69, 80.50, 116.55, 118.35, 118.88,123.74, 143.42, 148.11, 168.04; ESI-MS calculated for C_10_H_9_NO_3_ 191.18; found (M + H)^+^ 192.00.

*Preparation of 2-oxo-N-propylthiazolidine-4-carboxamide (2b)*

The desired product **2b** was obtained starting from the 2-oxo-N-thiazolidine-4-carboxilic acid (**2**) following the same procedure previously reported for compound **2a**, coupled to propylamine (0.402 g, 6.8 mmol). Yield 72%: mp 80.2-80.3°C; ^1^H NMR (400 MHz, DMSO-d_6_); δ 0.85 (t, 3H, CH_3_, J=7.04, J=7.43), 1.21-1.30 (m, 2H, CH_2_CH_3_), 3.04-3.09 (dd, 1H, CH_2_CH thiaz, J=6.65), 3.28-3.33 (dd, 1H, CH_2_CH thiaz, J= 8.61), 3.62 (t, 2H, CH_2_CH_2_CH_3_, J=6.65, J=7.04), 4.22 (t, 1H, CH_2_CHthiaz), 7.99 (s, 1H, NH), 8.23 (s, 1H, NH); ^13^C NMR (400 MHz, DMSO-d_6_) δ 14.1, 31.5, 32.8, 38.9, 56.9, 170.1, 173.7; ESI-MS calculated for C_7_H_12_N_2_O_2_S 188.25; found (M + H)^+^ 189.10.


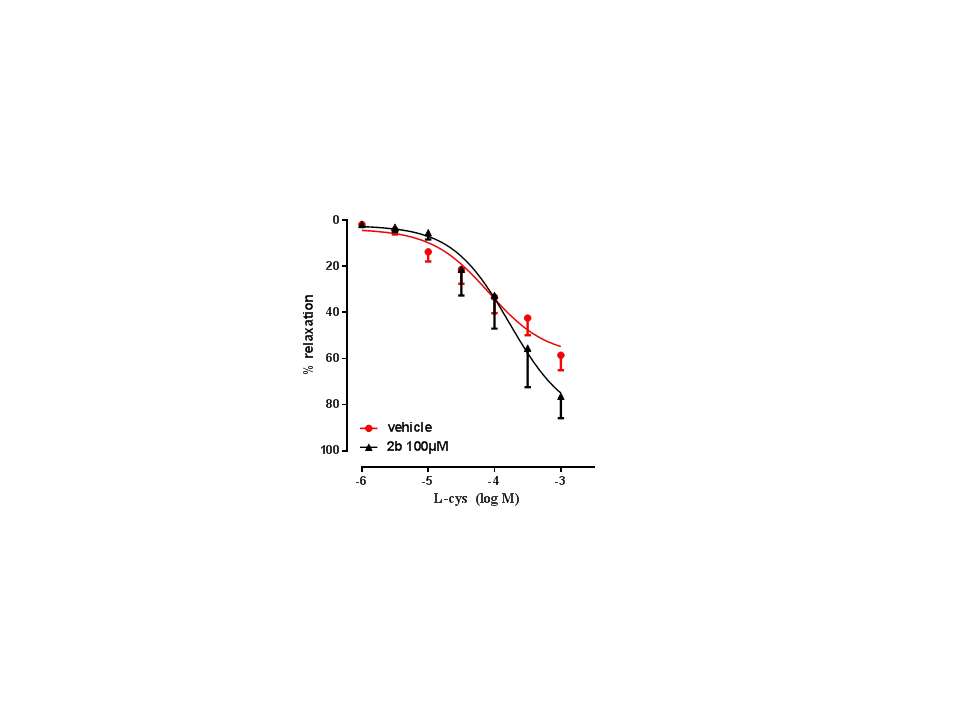


**Figure S3-** Incubation of aorta rings with 2b did not modify L-cysteine-induced vasodilatation

**Figure S4 -** Purification and characterization of the enzymes: A) SDS page; B) UV-vis absorption and far-UV CD spectra of the purified CBS (top) and CSE (bottom) enzymes. The absorption spectra are typical of these enzymes as shown by the typical PLP peak at 420 nm in CSE
and the resonance at 430 nm for CBS where both PLP and heme resonate.

**
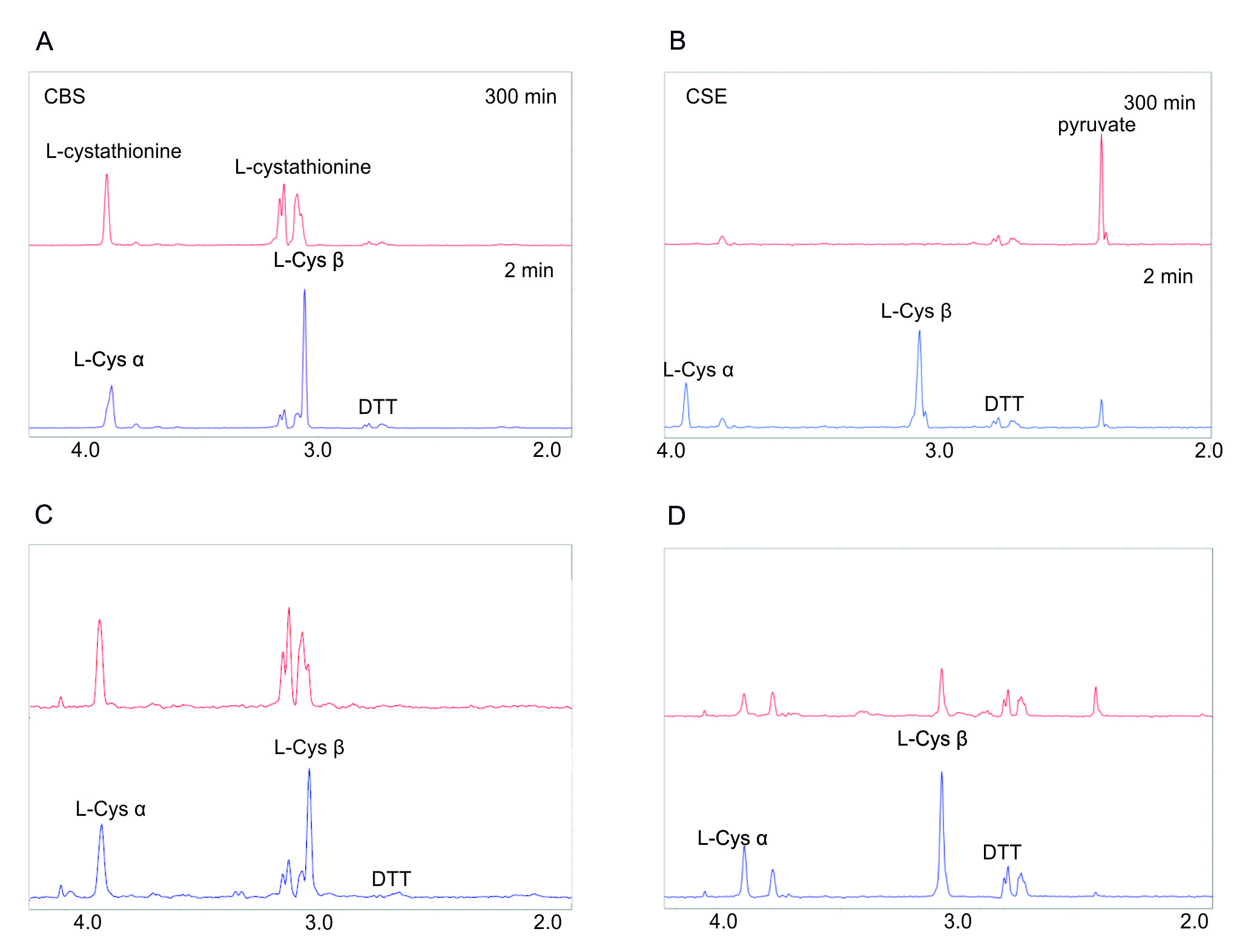
**

**Figure S5 - Enzymatic kinetics.** Real-time monitoring of CBS (**A**) and CSE (**B**) forward reaction by ^13^C filtered ^1^H NMR. The first (blue) and last (red) spectra are shown. The spectra show conversion of labelled L-Cys (mixed with unlabeled homocysteine as co-substrate for CBS) to L-cystathionine and pyruvate for CBS and CSE respectively. The first spectrum was obtained ~2 min after the addition of enzyme, and subsequently 59 spectra were collected over a 300 min period. The expected cystathionine resonances were not all detected, because of the unlabeled homocysteine contribution. ^13^C filtered ^1^H NMR spectra showing the time course in the presence of compound **2A** for CBS (**C**) and CSE (**D**).


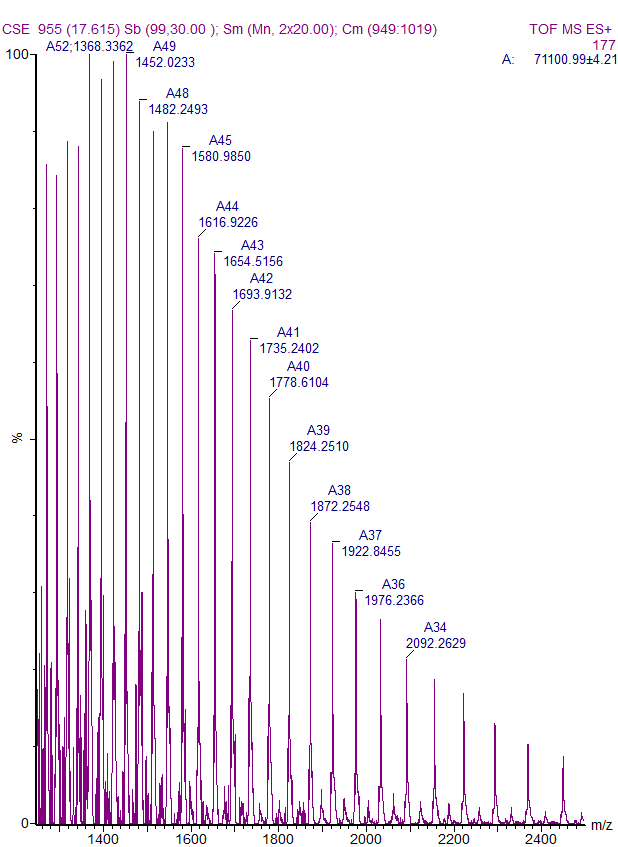

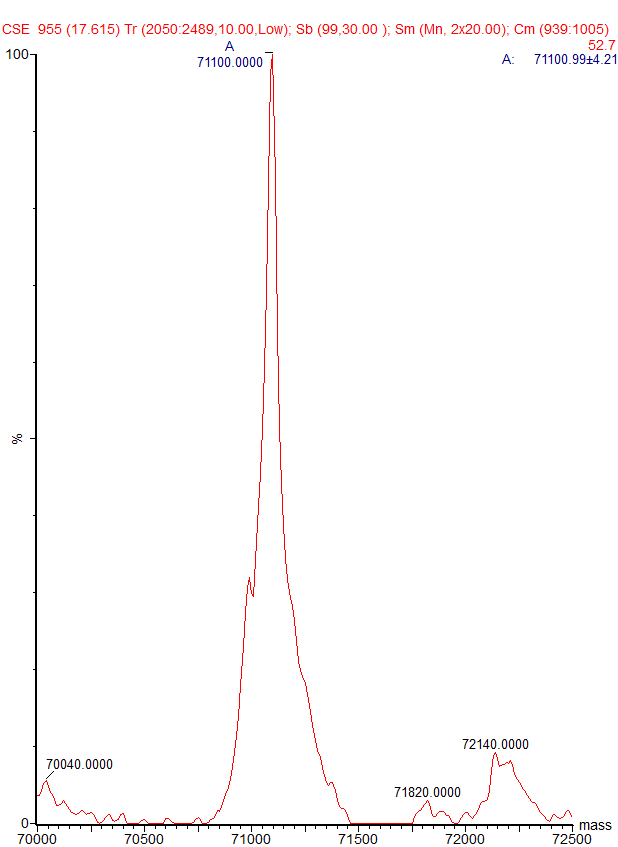

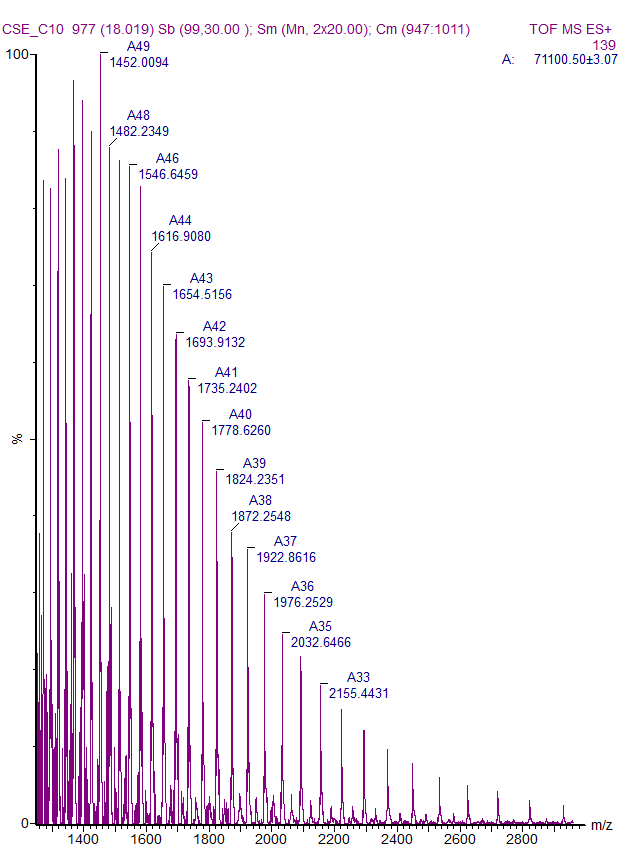

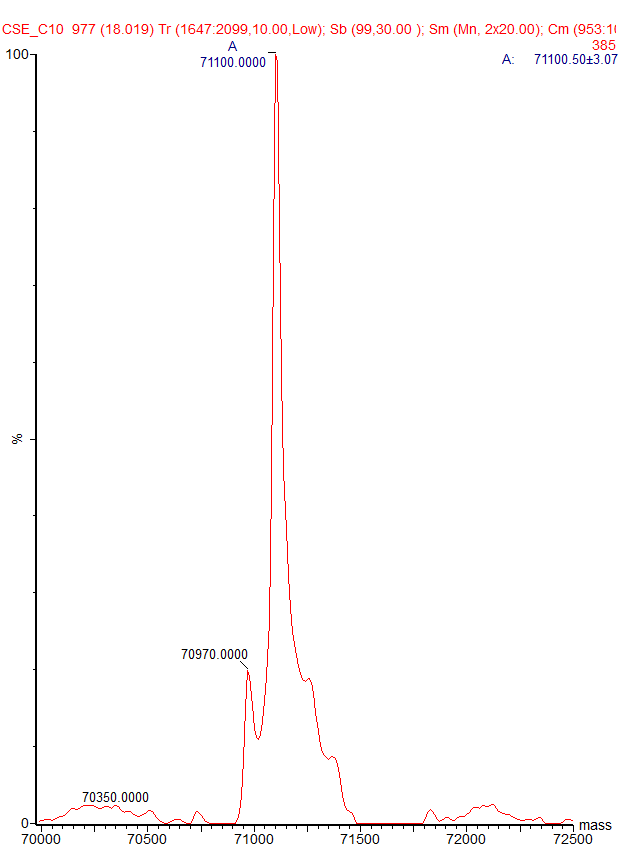

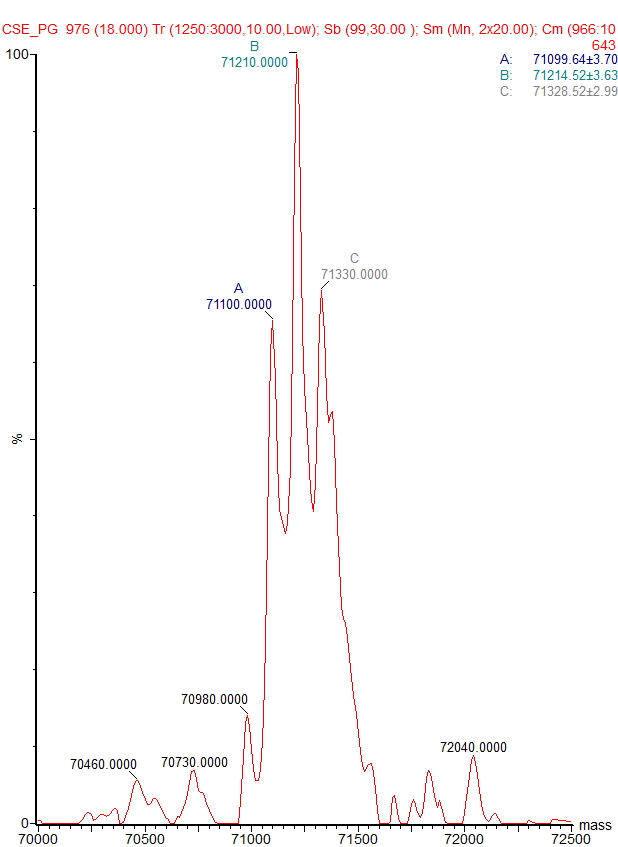

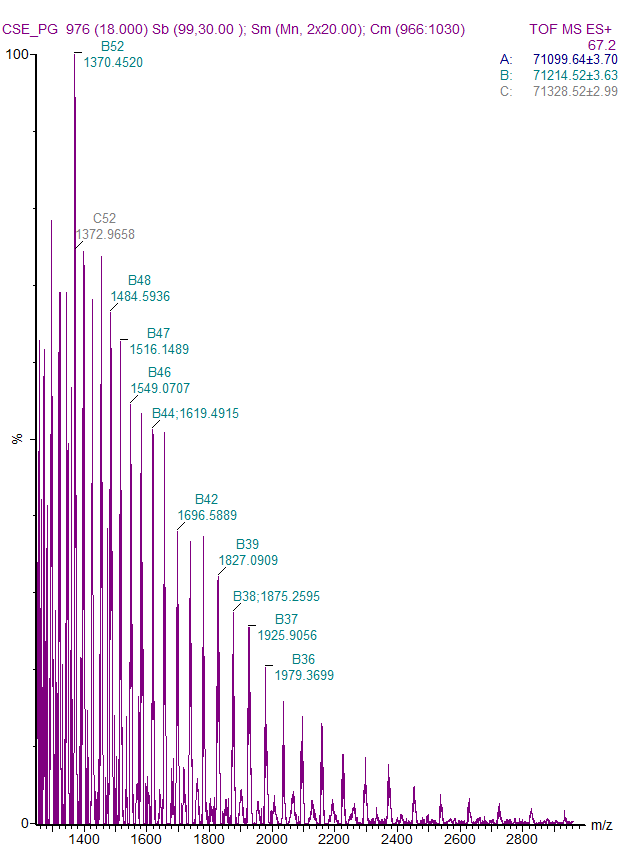


A

B

C

**Figure S6** – LC-MS spectra of (A) CSE (molecular weight 71100), (B) CSE-**2a** complex (molecular weight 71100) and (C) CSE-PAG complex (molecular weight 71210) which should be compared with the molecular weight of PAR of 113. These data conclusively show that **2a** is not a suicidal inhibitor.


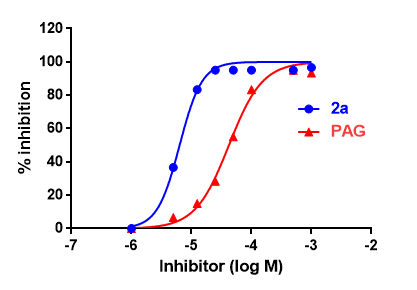


**Figure S7 –** IC_50_ calculated on the purified CSE using both PAG and compound 2a. It was obtained by separately making eight increasing concentrations of the two inhibitors from 0 to 0.001 M, adding L-Cys and incubating the reaction at 37 °C for 30 min. After this time all other reactives^17^ are added and measuring the absorbance at 670 nm after 20 min.

| **Vehicle** | E_max_ (%)  47.7±6.4 | n=3 |  |
| --- | --- | --- | --- |
| Compound **1** | 27.8±5.6 | n=3 |  |
| Compound **2** | 10.7±5.1 | n=3 | ** p<0.05 |
| Compound **3** | 15.7±5.0 | n=3 | * p<0.08 |
| Compound **4** | 10.7±5.3 | n=3 | ** p<0.05 |
| Compound **1a** | 26.7±6.7 | n=4 |  |
| Compound **2a** | 3.7±0.9 | n=3 | *** p<0.001 |
| Compound **3a** | 16.7±2.6 | n=4 | ** p<0.05 |
| Compound **4a** | 11.5±4.7 | n=3 | ** p<0.05 |

**Table S1 –** Emax values, which represent the maximal inhibitory effect, are given in percentages of the commercial and synthesized compounds.
